# Giving AI a ‘case library’ to diagnose battery failures

**DOI:** 10.1093/nsr/nwag483

**Published:** 2026-08-12

**Authors:** Callum Connor, Feng Lin

**Affiliations:** School of Engineering, Brown University, USA; School of Engineering, Brown University, USA

Tools to model and diagnose battery failure are essential to gauging and improving safety and reliability for energy storage systems. Machine learning (ML) and deep learning (DL) are two of the approaches to diagnosing battery failure. However, these tools are less than ideal for complex systems, like batteries. Diagnostics produced from ML and DL are specialized for the systems which they were trained on, require extensive labeled data for training, and are not easily interpreted [1]. Large language models (LLMs) have emerged as a powerful diagnostic tool relying primarily on experimental data inputs without the need for extensive labeled training data [2]. LLMs enable multi-task diagnosis through strong semantic understanding and their outputs are more easily interpreted than those of ML and DL [3,4].

LLMs can be further improved for complex systems such as batteries with methods like domain-specific fine-tuning (FT) and retrieval augmented generation (RAG). FT requires large amounts of data and struggles to adapt to new knowledge [5]. RAG retrieves data during generation, allowing for a more flexible model [6]. Knowledge-augmented generation (KAG) further improves upon RAG with knowledge structures that allow the LLM to make more informed decisions [7]. The ability of LLMs to reason and make informed decisions has led to their use in fields such as materials discovery.

In a recent paper of *National Science Review*, the authors present BattFailScholar (BFS) as a knowledge-augmented LLM for battery failure diagnostics [8]. The BFS LLM improves diagnostic accuracy with four core modules that make it specialized for battery diagnosis: a case-level battery failure knowledge graph (BF-KG, Fig. 1a), a failure-feature-aware case retrieval algorithm (Fig. 1b), a drift-aware result correction mechanism, and a pattern-based voting mechanism for quantitative risk assessment. This knowledge-augmented approach yields substantial improvements, with BFS significantly outperforming the base DeepSeek model across multiple diagnostic tasks (Fig. 1c). The case-level BF-KG organizes knowledge around experiential cases reported in the literature inspired by case-based reasoning. Ablation studies confirm that this design choice is doing the work: removing structured, feature-matched retrieval (in favor of random cases or raw text) hurts accuracy, while adding chain-of-thought prompting does not help. The BF-KG is built from >10 000 papers, collected with two-stage LLM-enabled extraction that leverages semantic understanding over rigid schemas. The BF-KG captures the relationships between material properties, multi-source signals, and failure chains to capture the pathways that lead to failure in a variety of systems. This algorithm selects reference cases from the knowledge graph on the basis of failure-feature similarity rather than surface-level matching. This algorithm enhances the precision of the LLM by matching features from the query case to those in the KG. This can better differentiate underlying factors that may manifest externally in a similar manner. The LLM is further improved with the drift-aware result correction mechanism. This modestly improves the diagnosis of less common failure modes, resolving challenging long-tail issues (Fig. 1d). The drift-aware result correction achieves this through label distribution shift and chemical system shift indicators, comparing the LLM’s innate reasoning with that of retrieved knowledge from the KG. To reduce overconfident outputs commonly produced by LLMs, BFS pulls common failure modes from a constructed prior knowledge base and matches them with predicted failure modes. Multi-case ensemble voting provides risk assessments for each failure mode to ensure that confidence scores are true to output accuracy.

**Figure 1.**
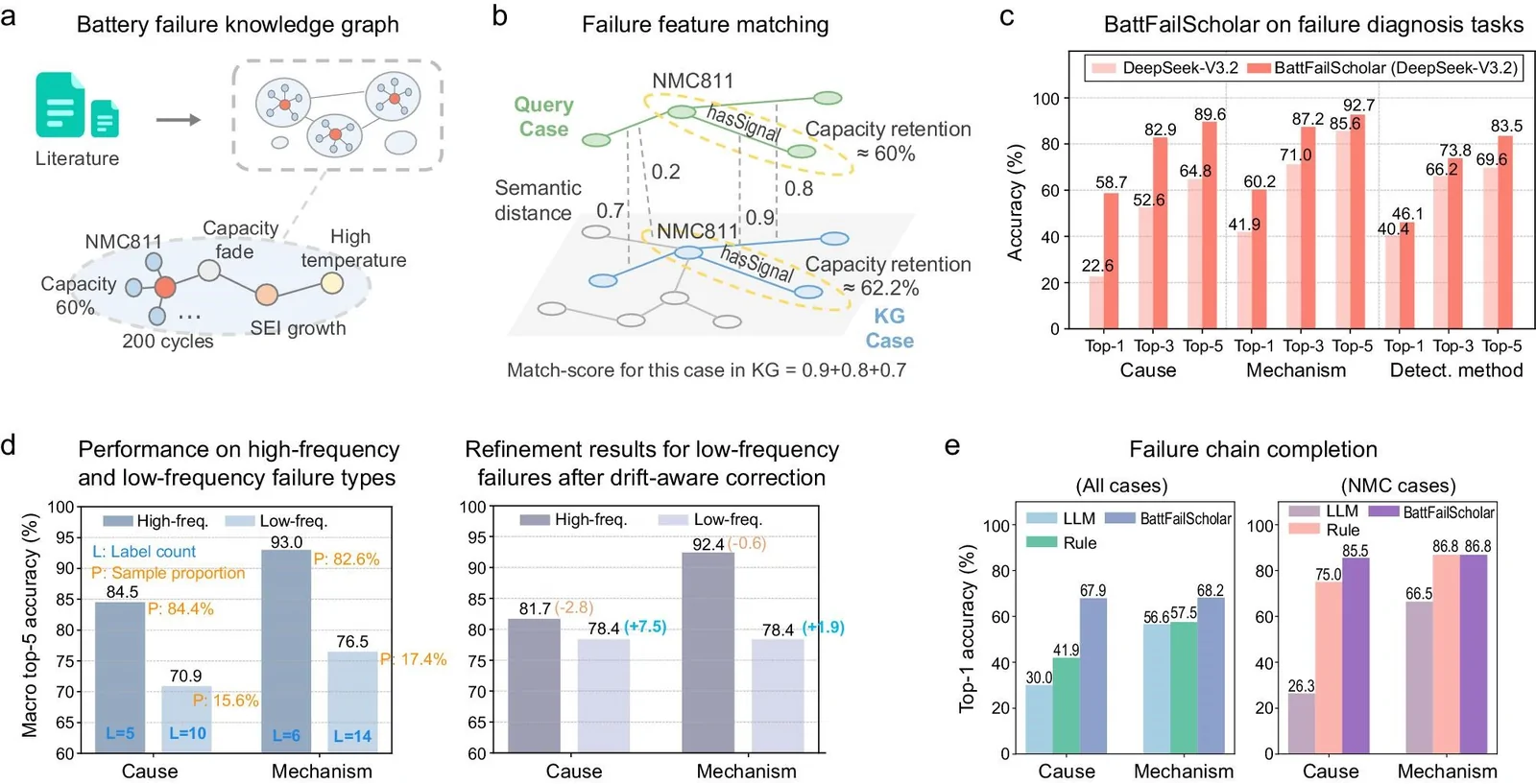
(a) A failure chain from the battery failure knowledge graph. (b) The failure-feature-aware case retrieval process. (c) Performance gain of BattFailScholar over DeepSeek. (d) Drift-aware correction results for low-frequency failures. (e) Results of inferring missing failure causes or mechanisms. Reproduced from Ref. [8] with permission.

BFS is compared to other LLMs and is shown to improve the accuracy of failure cause and mechanism diagnosis and recommendation of failure detection method. BFS shows a 19.7% average accuracy improvement over base LLMs across diagnostic tasks. Despite drawing on only a handful of retrieved cases, BFS matched or outperformed bidirectional long short-term memory (Bi-LSTM), bidirectional encoder representations from transformers (BRET), and graph neural network (GNN)-based models trained in a supervised fashion on the full case base. BFS is further validated for practical applications using real battery chemistries, showing diagnostic improvements for nickel manganese cobalt (NMC), lithium iron phosphate (LFP), and lithium cobalt oxide (LCO) systems. BFS is shown to be able to infer missing information with incomplete failure chains and determine missing failure causes or mechanisms in NMC batteries with enhanced accuracy (Fig. 1e). The model demonstrates its practicality by showing diagnostic improvements to the base LLM and shows accurate failure diagnostics of experimental thermal runaway data.

This study provides a powerful blueprint for combining the reasoning strengths of LLMs with structured domain knowledge. By creating a trustworthy AI assistant that delivers transparent, evidence-backed diagnoses, it points the way for knowledge-grounded scientific reasoning far beyond battery research.
